# Predictive biomarkers in the era of immunotherapy for gastric cancer: current achievements and future perspectives

**DOI:** 10.3389/fimmu.2025.1599908

**Published:** 2025-05-14

**Authors:** Fujing Sun, Xiaozhuo Gao, Wentao Wang, Xiaoyan Zhao, Jingdong Zhang, Yanmei Zhu

**Affiliations:** ^1^ Department of Pathology, Affiliated Cancer Hospital of Dalian University of Technology (Liaoning Cancer Hospital and Institute, Cancer Hospital of China Medical University), Shenyang, China; ^2^ Department of Gastric Surgery, Affiliated Cancer Hospital of Dalian University of Technology (Liaoning Cancer Hospital and Institute, Cancer Hospital of China Medical University), Shenyang, China; ^3^ Department of Gynecology, Affiliated Cancer Hospital of Dalian University of Technology (Liaoning Cancer Hospital and Institute, Cancer Hospital of China Medical University), Shenyang, China; ^4^ Graduate School, Dalian Medical University, Dalian, China; ^5^ Department of Gastroenterology, Affiliated Cancer Hospital of Dalian University of Technology (Liaoning Cancer Hospital and Institute, Cancer Hospital of China Medical University), Shenyang, China

**Keywords:** biomarker, gastric cancer, immune checkpoint inhibitor, immunotherapy, predictive value

## Abstract

Gastric cancer (GC) is one of the primary contributors to cancer-related mortality on a global scale. It holds a position within the top five most prevalent malignancies both in terms of occurrence and fatality rates. Immunotherapy, as a breakthrough cancer treatment, brings new hope for GC patients. Various biomarkers, such as the expression of programmed death ligand-1 (PD-L1), the microsatellite instability (MSI) status, tumor mutational burden (TMB), and Epstein–Barr virus (EBV) infection, demonstrate potential to predict the effectiveness of immunotherapy in treating GC. Nevertheless, each biomarker has its own limitations, which leads to a significant portion of patients continue to be unresponsive to immunotherapy. With the understanding of the tumor immune microenvironment (TIME), genome sequencing technology, and recent advances in molecular biology, new molecular markers, such as POLE/POLD1mutations, circulating tumor DNA, intestinal flora, lymphocyte activation gene 3 (LAG-3), and lipid metabolism have emerged. This review aims to consolidate clinical evidence to offer a thorough comprehension of the existing and emerging biomarkers. We discuss the mechanisms, prospects of application, and limitations of each biomarker. We anticipate that this review will open avenues for fresh perspectives in the investigation of GC immunotherapy biomarkers and promote the precise choice of treatment modalities for gastric cancer patients, thereby advancing precision immuno-oncology endeavors.

## 1 Introduction

Gastric cancer (GC) is prevalent worldwide, consistently ranking among the top five most lethal cancers in terms of both occurrence and fatality rates (1). National cancer statistics released by China’s National Cancer Centre show that in the year 2022, GC accounted for 358,700 new cases, with a mortality rate of 26.04 per 100,000 in China (2). Early GC can be effectively treated after surgery and the five-year survival rate can reach 90 to 100%. Nonetheless, owing to the subtle onset and rapid progression of the illness, almost all GC patients are diagnosed at an advanced stage. Traditional therapy for advanced GC typically involves chemotherapy. However, the clinical benefits of these therapies remain severely constrained. The median overall survival (OS) with conventional chemotherapy in advanced GC is merely 8 months (3).

**Figure 1:**
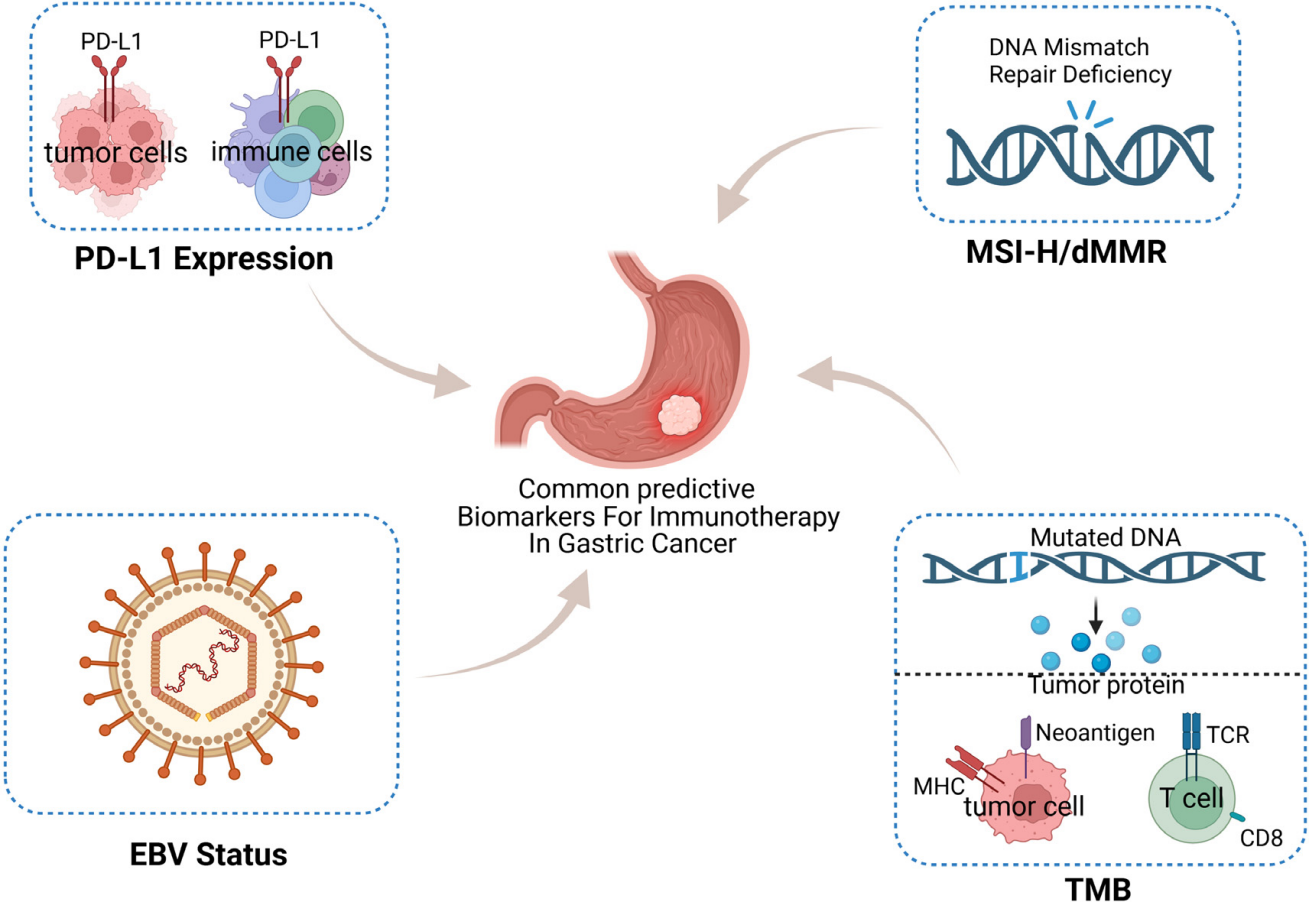
Classic biomarkers predicting the efficacy of neoadjuvant immunotherapy for gastric cancer (GC) include programmed death ligand 1 (PD-L1), the microsatellite instability (MSI) status, Epstein–Barr virus (EBV) infection and tumor mutational burden (TMB).

The advent of immune checkpoint inhibitors (ICIs) presents novel therapeutic avenues for solid tumors that have notably enhanced cure rates. This strategy finds strong support in the scientific community as it is based on the principle that ICIs primarily activate the body’s immune cells to target tumor cells, thereby acting to either remove these or reduce the tumor cell load (4). Currently, immune combination therapy is gradually becoming a treatment modality for GC, of which the most commonly used is the combination of chemotherapy and ICIs. Research has reported the safety and efficacy of ICIs in neoadjuvant therapy for GC (5); their application is gradually expanding from back-line to first-line therapy. Currently, biomarkers, including programmed death ligand 1 (PD-L1), the microsatellite instability (MSI) status, Epstein–Barr virus (EBV) infection and tumor mutational burden (TMB), are often applied clinically to predict immunotherapy efficacy (**Figure 1**). However, a considerable portion of patients fail to derive benefits from immunotherapy and encounter adverse effects linked with its use. Therefore, further research on biomarkers related to GC immunotherapy is particularly important for the accurate selection of appropriate cohorts for this treatment.

The current clinical evidence, mechanisms and limitations of existing biomarkers for predicting immunotherapy efficacy are discussed. This review aims to open avenues for new directions in research on biomarkers for GC immunotherapy.

## 2 Positive biomarkers

### 2.1 Programmed death ligand 1

PD-L1 is overexpressed in 25-65% of GC. In general, the PD-L1/PD-1 axis is recognized as an important factor for the poor prognosis of patients with different GC types. Firstly, PD-L1 expressed on GC cell surfaces inhibit anti-tumor immunity via multiple pathways. For example, over-expression of PD-L1 prevents the body’s immune system from recognizing GC cells; effector T cells are unable to target GC cells, leading to suppressing the anti-tumor immune response (6–8). Secondly, PD-L1 also binds to the epidermal growth factor receptor (EGFR) and activates it to promote GC progression (9). In addition to GC cells, PD-L1 is highly expressed on immune cell surfaces in the tumor immune microenvironment, including lymphocytes, neutrophils, macrophages, and mast cells (10).

#### 2.1.1 PD-L1 as an effective predictive marker

CPS (combined positive score) is defined as the ratio of PD-L1-positive tumor cells and immune cells to the total number of viable tumor cells. TAP (tumor area positivity) refers to the proportion of PD-L1-positive tumor and immune cell area relative to the total tumor area (viable tumor cells + stroma) (11). These two metrics are currently the most widely used quantitative indicators for assessing PD-L1 expression levels in clinical and research settings. While PD-L1 expression demonstrates a positive association with response to ICIs, the optimal predictive thresholds (CPS/TAP) for clinical benefit continue to be debated. For example, in the KEYNOTE-059 Cohort 1 trial, patients with PD-L1-positive (CPS ≥ 1) G/GEJ adenocarcinoma who received pembrolizumab monotherapy showed significantly higher objective response rates (ORR) and improved overall survival (OS) compared to PD-L1-negative patients (CPS < 1) (12). Promising follow-up data from the CheckMate-649 trial were presented at the 2024 ASCO GI Conference: patients with CPS ≥ 5 who received nivolumab in combination with chemotherapy continued to demonstrate clinically meaningful improvements in OS and progression-free survival (PFS) compared to those receiving chemotherapy alone in the randomized cohort (13). Additionally, for GC patients with TAP ≥ 5%, those treated with tislelizumab in combination with chemotherapy achieved longer median PFS, median OS, and duration of response (DoR) compared to those receiving placebo plus chemotherapy (14). Furthermore, in the KEYNOTE-062 trial, in patients with CPS ≥ 10, pembrolizumab monotherapy demonstrated significantly longer median OS compared to chemotherapy alone. However, when using a CPS cutoff of 1, no significant survival benefit was observed between the treatment groups (15). PD-1 inhibitors used in Phase III clinical trials are shown (12–20) (**Table 1**). Phase II and I clinical trials are shown (12, 21–38) (**Table 2**).

**Table 1:** Phase III trials of immunotherapy for G/GEJ adenocarcinoma.

Trial	Phase	Line	Ref.	Patient Selection	Number	Intervention	Immunotherapy agent	Clinical efficacy	Statistical evidence	Primary endpoint	≥3 Grade TRAEs
NCT02494583 (KEYNOTE-062)	III	First-line	(15)	CPS≥1,untreated, advanced G/GEJ adenocarcinoma	763	PEM + cisplatin + fluorouracil + capecitabine/P + chemotherapy (SAA)	PEM	OS (CPS≥1): 12.5 vs 11.1m; OS (CPS≥10): 12.3 vs 10.8m; PFS (CPS≥1): 6.9 vs 6.4m; median PFS (MSI-H): 11.2 VS 3.6m; ORR (MSI-H): 64.7%vs 36.8%; median OS (TMB≥10mut/Mb): 31.6 vs 13.4m; median PFS (TMB≥10mut/Mb): 11.1 vs 7.0m; ORR (TMB≥10mut/Mb): 55.6% vs 41.2%	OS (CPS≥1): HR 0.85, 95% CI 0.70-1.03, p=0.05; OS (CPS≥10): HR 0.85, 95% CI 0.62-1.17, p=0.16; PFS (CPS≥1): HR 0.84, 95% CI 0.70-1.02, p=0.04; median OS (TMB≥10mut/Mb): HR, 0.34; 95% CI: 0.14–0.82, p<0.05; median PFS (TMB≥10mut/Mb): HR, 0.52; 95% CI: 0.24–1.13, p<0.05; ORR (TMB≥10mut/Mb): p<0.05	OS and PFS	73% vs 69%
NCT02746796 (ATTRACTION-4)	III	First-line	(17)	HER2-negative, unresectable, advanced or recurrent G/GEJ adenocarcinoma	724	NIVO +(SOX or CAPOX)/P +(SOX or CAPOX)	NIVO	median PFS: 10.45 vs 8.34m; median OS: 17.45 vs 17.15m	median PFS: HR 0.68, 98.51% CI 0.51-0.90, p=0.0007; median OS: HR 0.90, 95% CI 0.75-1.08, p=0.26	PFS and OS	29% vs 25%
NCT03675737 (KEYNOTE-859)	III	First-line	(20)	locally advanced or metastatic HER2-negative G/GEJ adenocarcinoma	1579	PEM + (fluorouracil+cisplatin or capecitabine +oxaliplatin)/P + chemotherapy (SAA)	PEM	median OS: 12.9 vs 11.5m; median OS (CPS≥1): 13.0 vs 11.4m; median OS (CPS≥10): 15.7 vs 11.8m	median OS: HR 0.78, 95% CI 0.70–0.87, p<0.0001; median OS (CPS≥1): p<0.0001; median OS (CPS≥10): p<0.0001	OS	22% vs 18%
NCT02872116 (CheckMate-649)	III	First-line	(13)	unresectable, HER2-negative G/GEJ adenocarcinoma	1581	NIVO + (XELOX or FOLFOX)/(XELOX or FOLFOX)	NIVO	OS (CPS≥5): 14.4 vs 11.1m; PFS (CPS≥5): 7.7 vs 6.05m; median OS (MSI-H): 38.7 VS 12.3m; ORR (MSI-H): 55% vs 39%	OS (CPS≥5): HR 0.71, 98.4% CI 0.59-0.86,p<0.0001; PFS (CPS≥5): HR 0.68,98% CI 0.56-0.81, p<0.0001; median (MSI-H): HR 0.38, 95% CI 0.17-0.84; median (MSI-H): 95% CI 32-77	OS (CPS≥5) and PFS (CPS≥5)	59% vs 44%
NCT03777657 (Rationale 305)	III	First-line	(14)	TAP≥5% and unresectable locally advanced or metastatic GC/GEJ cancer	546	TIS + ICC/P + ICC	TIS	median OS: 17.2 vs 12.6m	median OS: HR 0.74; 95% CI 0.59-0.94; one-sided p=0.0056	OS	64.7% vs 62.9%
NCT02370498 (KEYNOTE-061)	III	Second-line	(19)	unresectable advanced G/GEJ adenocarcinoma	592	PEM/paclitaxel	PEM	median OS: 9.1 vs 8.3m; median PFS (CPS≥1): 1.5 vs 4.1m; median PFS (MSI-H): 17.8 vs 3.5m; ORR (MSI-H): 46.7% vs 16.7%	OS: HR 0.82, 95% CI 0.66-1.03, one-sided p=0.0421; median PFS (CPS≥1): HR 1.27, 95% CI 1.03-1.57	OS and PFS(CPS≥1)	14% vs 35%
NCT02625610 (JAVELIN Gastric 100)	III	Second-line	(18)	unresectable advanced or metastatic G/GEJ adenocarcinoma	499	avelumab/oxaliplatin plus a fluoropyrimidine	avelumab	24-month OS rate: 22.1% vs 15.5%; median OS (CPS≥1): 14.9 vs 11.6m	24-month OS rate: HR 0.91; 95% CI 0.74-1.11; p=0.1779	OS	61.3% vs 77.3%
NCT02267343 (ATTRACTION-2)	III	Third-line	(16)	unresectable recurrent or metastatic G/GEJ adenocarcinoma	493	NIVO/P	NIVO	median OS: 5.26 vs 4.14m; 12-month OS rate: 26.2% vs 10.9%	median OS: HR 0.63, 95% CI 0.51–0.78; p<0.0001	OS	10% vs 4%
NCT02335411 (KEYNOTE-059)-cohort1	III	Third-line	(12)	recurrent or metastatic G/GEJ adenocarcinoma	259	PEM	PEM	ORR: 11.6%; ORR (CPS≥1): 15.5% vs 6.4%; ORR (MSI-H): 57.1% vs 9.0%	NA	ORR	18%

SAA, same as above; OS, overall survival; PFS, progression-free survival; ORR, objective response rate; P, placebo; PEM, pembrolizumab; NIVO, nivolumab; TIS, tislelizumab; SOX, oxaliplatin + S-1; CAPOX, oxaliplatin + capecitabine; FOLFOX, oxaliplatin + fluorouracil + folinic acid; ICC, oxaliplatin + capecitabine/cisplatin + 5-fluorouracil.

**Table 2:** Phase II and I trials of immunotherapy for G/GEJ adenocarcinoma.

Trial	Phase	Line	Ref.	Patient Selection	Number	Intervention	Immunotherapy agent	Clinical efficacy	Statistical evidence	Primary endpoint	≥3 Grade TRAEs
NCT02335411 (KEYNOTE-059)-cohort2	II	First-line	(12)	recurrent or metastatic G/GEJ adenocarcinoma	25	PEM + cisplatin + (5-fluorouracil or capecitabine)	PEM	ORR: 60%; ORR (CPS≥1): 68.8% vs 37.5%	NA	ORR	76%
NCT02335411 (KEYNOTE-059)-cohort3	II	First-line	(12)	CPS≥1 and recurrent or metastatic G/GEJ adenocarcinoma	31	PEM	PEM	ORR: 25.8%	NA	ORR	23%
NCT03382600 (KEYNOTE-659)-cohort1	IIb	First-line	(23)	CPS≥1 and advanced G/GEJ cancer	54	PEM + SOX	PEM	ORR: 72.2%; ORR (CPS ≥1 to <10): 73.9% ; ORR (CPS≥10): 71.0%	NA	ORR	59.30%
NCT03382600 (KEYNOTE-659)-cohort2	IIb	First-line	(23, 31)	CPS≥1 and advanced G/GEJ cancer	46	PEM + SP	PEM	ORR: 80.4%	NA	ORR	78.30%
NCT02918162	II	First-line	(33)	resectable G/GEJ adenocarcinoma	36	PEM + XELOX	PEM	pCR rate: 20.6%	NA	pCR	57.10%
NCT04065282	II	First-line	(35)	resectable G/GEJ adenocarcinoma stage cT3-4NanyM0	36	SIN + XELOX	SIN	pCR rate: 19.4%; MPR rate: 47.2%	NA	pCR and MPR	13.90%
ChiCTR2000030414	II	First-line	(25)	locally advanced GC	30	SIN + XELOX	SIN	pCR: 42.1% (CPS≥1) vs 18.2% (CPS<1); MPR: 78.9% (CPS≥1) vs 36.4% (CPS<1); pCR: 66.7% (CPS≥5) vs 19.0% (CPS<5); MPR: 100.0% (CPS≥5) vs 47.6% (CPS<5)	pCR (CPS≥1 vs <1): p=0.246; MPR (CPS≥1 vs <1): p=0.047; pCR (CPS≥5 vs <5): p=0.018; MPR (CPS≥5 vs <5): p=0.011	pCR and MPR	9.90%
NCT03421288(Dante)	II	First-line	(26)	G/GEJ adenocarcinoma with a clinical stage ≥cT2 and/or cN1	295	ATZ + FLOT/FLOT	ATZ	pCR rate: 24% vs 15%; pCR rate (CPS≥10): 33% vs 12%	pCR rate: one-sided p=0 .032	pCR	69% vs 66%
NCT04250948(NEOSUMMIT-01)	II	First-line	(36)	resectable G/GEJ cancer clinically staged as cT3-4aN + M0	108	toripalimab + (SOX or XELOX)/SOX or XELOX	toripalimab	pCR: 22.2% vs 7.4%	pCR: p=0.03	pCR	NA
NCT03399071(ICONIC)	II	First-line	(28)	early stage OGA	34	avelumab+FLOT	avelumab	pCR rate: 15%	NA	pCR	NA
NCT04006262(NEONIPIGA)	II	First-line	(27)	locally advanced resectable dMMR/MSI-H G/GEJ adenocarcinoma	32	NIVO + IPI	NIVO and IPI	pCR rate: 58.6%	NA	pCR	19.00%
NCT03448835(PANDA)	II	First-line	(21)	resectable G/GEJ adenocarcinoma	21	ATZ + docetaxel + oxaliplatin + capecitabine.	ATZ	pCR rate: 70%; MPR rate: 45%	NA	pCR and MPR	10%
NCT03939962	II	First-line	(22)	resectable locally advanced G/GEJ adenocarcinoma	42	camrelizumab + FLOFOX	camrelizumab	pCR rate: 9.1%	NA	pCR	51%
NCT03631615(Neo-PLANET)	II	First-line	(38)	resectable locally advanced G/GEJ adenocarcinoma	36	camrelizumab + capecitabine + oxaliplatin	camrelizumab	pCR rate: 33.3%	NA	pCR	69.40%
NCT04890392(WuhanUHGI001)	II	First-line	(37)	resectable locally advanced HER2-negative G/GEJ cancer	32	TIS + SOX	TIS	MPR: 53.1%	NA	MPR	65.60%
NCT03469557	II	First-line	(34)	locally advanced or metastatic G/GEJ adenocarcinoma	15	TIS + XELOX	TIS	ORR: 46.7%; median DoR: 12.8m; DCR: 80%	NA	ORR,DoR and DCR	
NCT02628067 (Keynote-158)	II	Second-line	(30)	unresectable and advanced MSI-H/dMMR GC	24	PEM	PEM	ORR: 45.8%	NA	ORR	15.00%
NCT02915432	Ib/II	Second-line	(32)	unresectable and advanced G/GEJ adenocarcinoma	76	toripalimab/toripalimab + oxaliplatin + capecitabine	toripalimab	ORR: 12.1% vs 66.7%	NA	ORR	22.4% vs 38.9%
NCT01848834 (KEYNOTE-012)	Ib	Third-line	(29)	CPS≥1 and recurrent or metastatic G/GEJ adenocarcinoma	39	PEM	PEM	ORR: 33%	NA	ORR	13%
NCT01928394 (CheckMate-032)	I/II	Third-line	(24)	locally advanced or metastatic gastric, esophageal, or GEJ adenocarcinoma	160	NIVO3/NIVO1 + IPI3/NIVO3 + IPI1	NIVO and IPI	ORR: 12% vs 24% vs 8%; ORR (MSI-H): 29% vs 11%, 50% vs 19%, 50% vs 18%	NA	ORR	17% vs 47% vs 7%

OGA, oesophagogastric adenocarcinoma; pCR, pathological complete response; MPR, major pathological response; EFS, event-free survival; OS, overall survival; DCR, disease control rate; DoR, duration of response; P, placebo; PEM, pembrolizumab; NIVO, nivolumab; IPI, ipilimumab; SOX, oxaliplatin + S-1; SP, S-1 + cisplatin; TIS, tislelizumab; SIN, sintilimab; XELOX, capecitabine + oxaliplatin; FLOT, 5-fluorouracil + leucovorin + oxaliplatin + docetaxel ; ATZ, atezolizumab; FOLFOX, oxaliplatin + fluorouracil + folinic acid.

Initial studies have demonstrated the potential predictive value of PD-L1 expression thresholds (CPS ≥ 1, CPS ≥ 5, TAP ≥ 5%, and CPS ≥ 10) for immunotherapy efficacy. However, due to variations in patient cohort sizes and differences in study endpoint definitions across clinical trials, further large-scale prospective studies are needed to optimize these cutoff values and improve their predictive accuracy and consistency.

#### 2.1.2 Mechanism

The mechanism by which PD-1 inhibitors can effectively kill tumor cells is closely related to the TIME. Wei et al. found that patients with GC treated with chemotherapy combination and neoadjuvant PD-1 inhibitors had increased CD8^+^ T-cell counts and M1/M2 macrophage ratios (39). Using a GC mouse model, Tang et al. demonstrated that mice achieving a major response to neoadjuvant PD-1 inhibitors plus chemotherapy exhibited significant infiltration of anti-tumor immune cells, including CD8^+^ CD44^+^ CD62L^-^ effector T cells and CD8^+^ T cells, along with a high M1/M2 macrophage ratio (40). Avgustinovich et al. found that GC patients with CPS ≥ 10 who were sensitive to neoadjuvant immune-combination chemotherapy showed activation of autophagy, which is involved in the activation of tumor immunity (41, 42). Autophagy can not only directly down-regulate PD-L1 through the p62/SQSTM1-NF-κB pathway (43), but also down-regulate PD-L1 by reducing the expression of histone deacetylase (HDAC) (44), participating in the activation of tumor immunity. Therefore, activation of autophagy may be an important factor for their sensitivity to immunotherapy.

While most clinical trials have demonstrated that gastric/gastroesophageal junction cancer (G/GEJC) patients with high PD-L1 expression benefit from immunotherapy, a minority of studies have failed to achieve satisfactory outcomes. In KEYNOTE-061 (CPS ≥ 1), pembrolizumab failed to improve OS as second-line therapy for advanced G/GEJC than paclitaxel (19). Similarly, avelumab (JAVELIN Gastric 100) (18)and nivolumab (ATTRACTION-4) (17)showed no OS/PFS benefits over chemotherapy in later-line settings. Therefore, it is essential to investigate the mechanisms underlying resistance to ICIs in GC.

**Figure 2:**
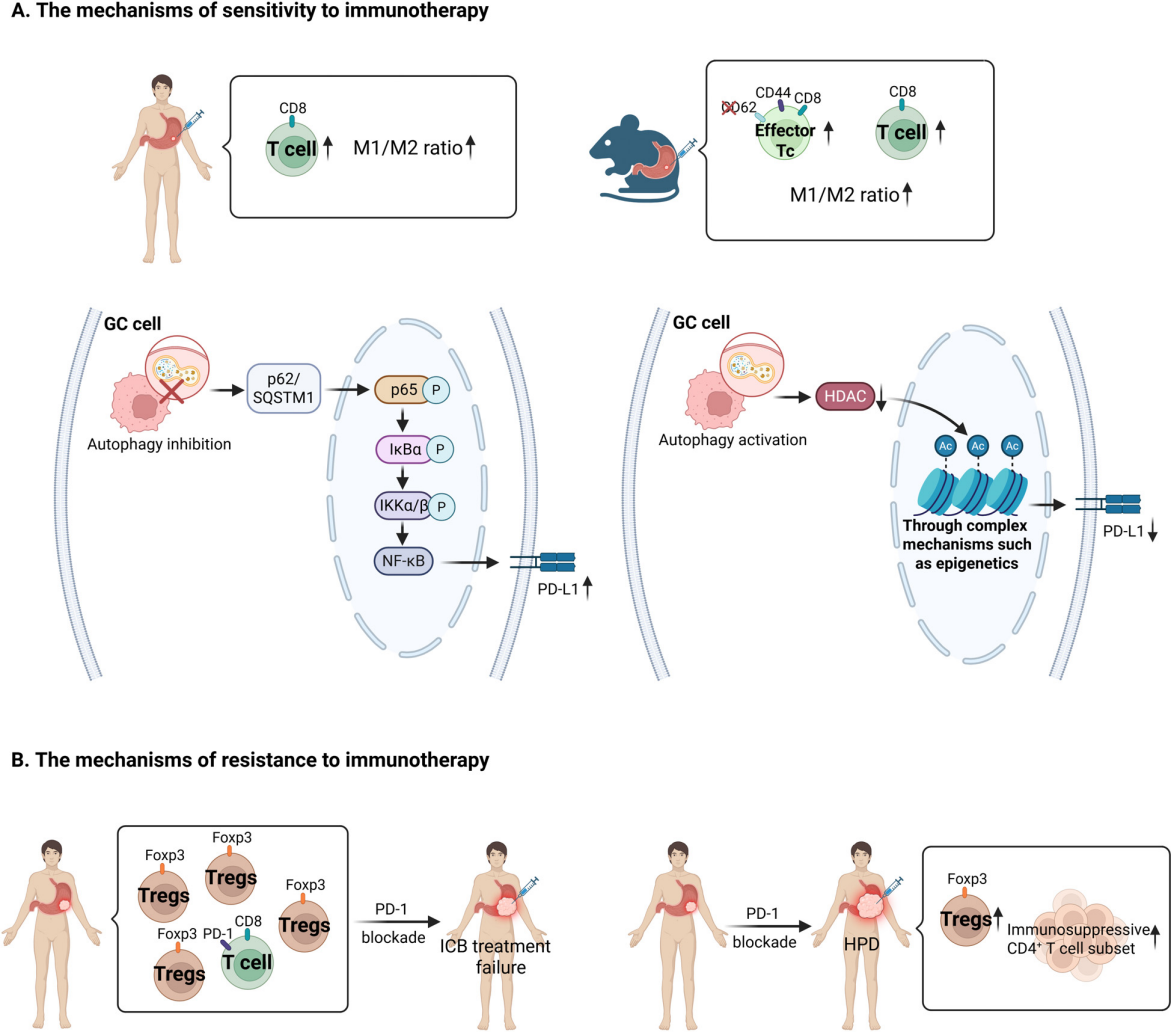
A summary diagram illustrating PD-L1-related immune sensitivity and resistance mechanisms in GC. **(A)** The mechanisms of sensitivity to immunotherapy. A favorable response to neoadjuvant PD-1 inhibitor combined with chemotherapy is associated with increased infiltration of CD8^+^ T cells and CD8^+^ CD44^+^ CD62L^-^ effector T cells, as well as an elevated M1/M2 macrophage ratio. Autophagy regulates PD-L1 expression in cancer cells. Autophagy inhibition leads to upregulation of p62/SQSTM1, which translocates into the nucleus and then activates NF-κB signaling pathway, ultimately upregulating PD-L1 expression. Autophagy activation attenuates PD-L1 expression via histone deacetylase (HDAC) downregulation, with subsequent complex mechanism such as epigenetic remodeling. **(B)** The mechanisms of resistance to immunotherapy. Low pre-treatment levels of CD8^+^ PD-1^+^ T-cell infiltration and high levels of Treg cell infiltration may contribute to immunotherapy failure. Patients exhibiting hyper-progressive disease (HPD) following immunotherapy demonstrate increased Treg cell infiltration and expansion of immunosuppressive CD4^+^ T-cell subsets within tumor tissues.

There is little exploration on the mechanism of resistance to PD-1 inhibitor, and it has been clear that differences in immune cells in TME are related to PD-1 inhibitor resistance. In high-CPS G/GEJ adenocarcinoma patients failing to respond to therapy, Verschoor et al. found a low degree of CD8^+^ PD-1^+^ T-cell infiltration, as well as a TME with high regulatory T (Treg) cell infiltration, which may be the cause of immune checkpoint blocking (ICB) treatment failure (21). Of course, there are even worse cases. Approximately 10% of patients with GC receiving PD-1 blockade progress rapidly (45), to hyper-progressive disease, which is related to Treg cell proliferation in tumor tissue (46), an increase in immunosuppressive CD4^+^ T cell subset that hinders effective anti-tumor immunity (47–52). In addition to differences in immune-infiltrating cells in TME, the spatiotemporal heterogeneity of PD-L1 expression (53, 54), the accuracy of PD-L1 assessment, including sample collection and assay methodology (55), and the complexity of the immunotherapy mechanism (56) can lead to the failure of PD-L1 to effectively predict immunotherapy efficacy. Therefore, more research is required to explore immunotherapy resistance mechanisms in GC patients and identify suitable alternative strategies. Current studies have identified USP7 inhibitors, which are new anti-proliferative agents that inhibit GC cell proliferation and down-regulate PD-L1 expression both *in vivo* and *in vitro* (57). Thus, with more research, more targets could be developed to overcome immunotherapy-related drug resistance (**Figure 2**).

### 2.2 Microsatellite instability–high/deficient mismatch repair

Microsatellite instability–high/deficient mismatch repair (MSI-H/dMMR) represents a specific subtype of tumor that accounts for 8–10% of GC characterized by high tumor immunogenicity and dense immune cell infiltration (58, 59). Given relatively low MSI-H GC incidences, no targeted Phase III clinical trials have been conducted. As ICIs have revolutionized the therapy of MSI-H/dMMR malignancies, their potential in neoadjuvant therapy is receiving more attention. MSI-H GC, characterized by defective DNA mismatch repair, exhibits a high frequency of somatic mutations, leading to the generation of abundant neoantigens (60). These neoantigens can activate robust anti-tumor T-cell responses and are associated with increased infiltration of anti-tumor immune cells within the tumor microenvironment, thereby rendering MSI-H tumors particularly sensitive to ICIs (61).

#### 2.2.1 Advanced unresectable G/GEJ adenocarcinoma

In the KEYNOTE-062 trial, a subgroup analysis of 7% of patients with MSI-H G/GEJ adenocarcinoma (15) showed that ORR and PFS outcomes in patients receiving pembrolizumab alone and pembrolizumab combined chemotherapy were significantly better than those receiving chemotherapy alone (62). An analysis of 3% of 1581 advanced unresectable HER2-negative GC/GEJC patients (with MSI-H status) in the CheckMate649 study showed that regardless of PD-L1 expression status, MSI-H subgroup patients had higher OS benefits when compared to patients who had chemotherapy alone (63). KEYNOTE-061 is a phase III clinical trial where randomized patients with advanced unresectable G/GEJC were evaluated in terms of pembrolizumab effectiveness and safety as a second-line treatment. An analysis of 27 patients (5.3%) with MSI-H status showed that (62) when compared to the chemotherapy group, median PFS and ORR outcomes in the pembrolizumab monotherapy group were significantly improved. By comparing early survival curves in patients with MSI-H tumors treated with pembrolizumab with patients with MSI-H tumors treated with chemotherapy alone in KEYNOTE-061 and KEYNOTE-062 trials, respectively (15), early pembrolizumab administration in GC patients with the MSI-H subtype gained benefits. Marabelle et al., examined the efficacy and safety profiles of second-line pembrolizumab in 223 patients with advanced MSI-H/dMMR cancer and failed to respond to chemotherapy, of whom, 10.3% had GC. The ORR in patients with MSI-H/dMMR GC was 45.8%, which was higher than the overall 34.3% ORR; the number of patients with complete responses (4) was second only to endometrial cancer. More importantly, tumor responses were long-lasting, with > 75% of respondents showing durable responses of ≥ 24 months in Kaplan–Meier analyses (30). In terms of third-line treatment results in patients with MSI-H G/GEJ adenocarcinoma: 7/259 patients registered in the KEYNOTE-059 cohort 1 had MSI-H GC and received pembrolizumab monotherapy (62). When compared to non-MSI-H patients, the ORR was higher in these seven patients (57.1% versus 9.0%) (64). One patient in Cohort 3 had an MSI-H tumor and experienced a partial response (12). In the CheckMate032 trial, the proportion of patients with MSI-H status (28%) was higher in the NIV03 group when compared to any combination group (NIVO1 + IPI3: MSI-H, 9% or NIV03 + IPI1: MSI-H, 8%). This was possibly why the median OS across groups was not very different. Also, by comparing MSI-H with non-MSI-H patients in groups, the results found that whether it is ORR, 12-month OS rate, or 18-month OS rate, the values of MSI-H patients are better (24).

#### 2.2.2 Locally progressive dMMR/MSI-H G/GEJ adenocarcinoma (neoadjuvant)

Pembrolizumab, ipilimumab, nivolumab, and atezolizumab also demonstrated favorable clinical activity in patients with resectable dMMR/MSI-H G/GEJ adenocarcinoma. Building on previous KEYNOTE-062 data, Liu et al., incorporated pembrolizumab into neoadjuvant chemotherapy to treat six patients with advanced non-metastatic MSI-H GC, and all patients showed good pathological responses (65). In another Phase II clinical trial (NCT02918162), three patients with advanced resectable G/GEJ who received pembrolizumab in combination with chemotherapy had an MSI-H status (33). One patient achieved pathological complete response (pCR). Based on previous CheckMate649 data, the recent NEONIPIGA trial treated patients with locally advanced dMMR/MSI-H G/GEJ adenocarcinoma with neoadjuvant ipilimumab + nivolumab during the perioperative period (27). Of the 29 patients who received an R0 resection, 17 (58.6%) achieved pCR (pathological T0N0) and had no unexpected immune-related adverse events, postoperative morbidity, or death. The other four with locally advanced resectable MSI-H G/GEJ adenocarcinoma who received neoadjuvant ipilimumab + nivolumab had pathological reactions, three achieved pCR, and the other was confirmed with a reduced the tumor-node-metastasis (TNM) stage (27). In the DANTE study (26), the pCR rate in patients with MSI-H GC treated with the 5-fluorouracil + leucovorin + oxaliplatin + docetaxel (FLOT) and atezolizumab (ATZ) (63%) combination was significantly better versus patients treated with FLOT (27%). In the PANDA trial, patients with treatment-naïve resectable G/GEJ tumors (n = 20) with neoadjuvant treatment with atezolizumab in combination with chemotherapy and subsequent surgical resection. Both dMMR patients in this trial achieved pCR (21).

A number of other PD-1 inhibitors are available as combinations of neoadjuvant chemotherapy for people with resectable G/GEJ adenocarcinoma with an MSI-H/dMMR status. Two (5.6%) patients with a dMMR were included in a phase II clinical trial (NCT04065282) aimed to evaluate sintilimab plus chemotherapy (CapeOx) as a neoadjuvant therapy protocol for people with advanced resectable G/GEJ adenocarcinomas (35). In one case their CPS = 68; this patient received a pCR. Another individual, whose PD-L1 expression status was unknown, did not exhibit major pathological response (MPR). A phase II trial investigating the efficacy of camrelizumab + FLOFOX (oxaliplatin+calcium folinate+5-fluorouracil) (NCT03939962) included cases with locally advanced resectable GC/GEJ adenocarcinoma, including one patient with an MSI-H status who achieved a pCR (66).

Limited research exists on the mechanisms of efficacy of immunotherapy for MSI-H tumors. One study applied an exploratory multiple fluorescence analysis to show that in patients with MSI-H, immune-combination chemotherapy increased tumor CD3^+^ and CD8^+^ T-cell densities, and that the degree of a pathological response was associated with increased clustering of CD3^+^ cells to panCK^+^ cells (33). Kwon et al., reported that increased PD-1^+^ CD8^+^ T cells were associated with lasting therapeutic benefit in patients with MSI-HGC (67). Additionally, patients with up-regulated PD-L1 expression in MSI-H GC showed a good clinical prognosis (68–72). But approximately 50% of patients with MSI-H tumors also exhibit intrinsic resistance to PD-1 inhibitors. Ongoing research is actively investigating primary resistance to ICIs in people with advanced MSI-H GC/GEJC. Evidence from the KEYNOTE-059, -061, and -062 trials indicates that the loss of heterogeneity in mismatch repair enzymes within tumors may underlie diminished response to treatment with pembrolizumab alone (73). Future studies should integrate multi-omics approaches (e.g., single-cell sequencing, spatial transcriptomics) with functional experiments to systematically uncover immunotherapy resistance mechanisms in MSI-H GC.

### 2.3 EBV

Epstein-Barr virus–associated GC (EBVaGC) accounts for 2-10% of all GC cases (74). Epstein-Barr virus (EBV) can cause a local immune response (75, 76). Analysis of tumor genomic profiles shows that EBV^+^ tumors are often microsatellite-stable (MSS) (77). Evidence exists of a low tumor mutational burden and stronger immune infiltration in EBV^+^ tumors compared to MSI-H tumors. In addition, EBV^+^ tumors showed higher expression of immune checkpoint pathway (PD-1, CTLA-4) genes and higher infiltration of lymphocytes (e.g., follicular helper T cells and CD8^+^ T cells (78)) in their RNA sequence data compared to MSS tumors. Therefore, the use of anti-PD-1 and anti–CTLA-4 monoclonal antibodies in a neoadjuvant setting is possible for patients with EBV^+^ GC.

#### 2.3.1 Unresectable advanced G/GEJ adenocarcinoma

A phase II trial by An et al. involved 47 treatment-naïve patients with advanced gastroesophageal adenocarcinoma (GEA) who were treated with first-line pembrolizumab in combination with chemotherapy, including two (4.3%) EBV^+^ patients who achieved a CR and PR, respectively. Bai et al. analyzed 66 people with unresectable GC managed with ICB at a proficient mismatch repair and showed that compared to EBV^-^ patients, 22 EBV^+^ patients exhibited a better ORR. Survival analysis revealed that EBV^+^ cases had better PFS and OS compared to EBV^-^ cases. The EBV status is a strong prognostic factor for PFS in patients with GC after ICB (79). What is clear is that, as with performance in neoadjuvant therapy, patients with advanced EBVaGC have varying rates of efficacy for immunotherapy. Based on the results of the above clinical trials, re-screening of patients with EBVaGC before applying immunotherapy appears to be crucial.

Kim et al. analyzed patients with metastatic GC treated with second-line pembrolizumab monotherapy following first-line chemotherapy failure. It was found that six patients who were EBV^+^ GC had an ORR of 100% and were all positive for PD-L1 expression (CPS ≥ 1) (73). Wang et al. conducted a phase Ib/II clinical trial aimed at assessing the safety and effectiveness of toripalimab for treating advanced GC. Four EBV^+^ patients participated in this trial, of which one patient with a positive CPS ≥ 1 achieved a PR; the other three EBV^+^ patients who did not achieve a PR were all negative for PD-L1 expression (32). These results demonstrate that testing for PD-L1 in patients with EBVaGC is necessary and that patients with positive CPS values (CPS ≥ 1) may be better suited for immunotherapy.

#### 2.3.2 Untreated, resectable locally advanced EBVaGC

The Neo-PLANET phase II study evaluated first-line neoadjuvant camrelizumab in combination with chemotherapy to 36 patients with resectable T3-4N+M0 G/GEJ adenocarcinoma. One of the patients with EBV^+^ had 80% residual tumor cells (38). One of the six patients in the PANDA trial who did not respond to neoadjuvant atezolizumab was EBV^+^, and this patient had up to 60-70% residual tumor cells (21). The expression of PD-L1 in the two EBV^+^ patients in these two clinical trials is undetermined. In an analysis of 77 patients with locally advanced GC who underwent neoadjuvant therapy followed by D2 radical surgery, three EBV^+^ patients were in the PD-1 blockade plus chemotherapy group; one patient with a CPS of 70 achieved a pCR, and two others with CPSs of 10 and 1 achieved an MPR (80). In view of these results, Wei et al. included 159 cases with EBVaGC for PD-L1 immunohistochemical analysis in order to explore the effect of PD-L1 expression in EBVaGC on patient outcomes and prognosis. Patients with CPS ≥ 1 had a greater objective efficacy rate than those who were negative (*P* = 0.001); ORR values of 83.3% and 100.0% were found for patients with CPS ≥ 10 and CPS ≥ 50, respectively. In terms of prognosis, cases with CPS ≥ 1 showed greater survival (*P* ≤ 0.001) and longer disease-free survival compared to cases with CPS < 1 (81). These results suggest that PD-L1 is an effective marker for screening the benefit of immunotherapy in EBVaGC.

In addition to PD-L1, Bai et al. analyzed differences in the TME and genomic characteristics distinguishing patients with EBVaGC who responded to ICB therapy from those who did not respond to screen people for EBVaGC immunotherapy. In the group of responders, a notably higher frequency of *SMARCA4* gene mutations were found compared to the non-responder group, which may be linked with higher TMB levels in cases with *SMARCA4* variants compared to those with wild-type *SMARCA4* (79). These findings indicate that TMB and mutations in *SMARCA4* may serve as promising predictive biomarkers for the effectiveness of ICB therapy in EBVaGC.

Studies have also shown that EBV^+^ GC cells not only promoted *CD274* (the gene that encodes PD-L1) amplification by activating the IRF3/CD274 axis, but also up-regulated constitutive PD-L1 expression (82). EBV infection stimulated increased interferon (IFN)-γ levels in GC and induced adaptive PD-L1 expression (82–84). In addition, Bai et al. analyzed the TME differences between ICB responders and non-responders in EBVaGC and found that the level of intratumoral CTLA-4 and the density of T-cell immunoglobulin-3 (TIM-3)^+^ cells exhibited a notable elevation in the group of patients who did not respond to ICB therapy, compared to those who showed a positive response. The above studies can explain to some extent the mechanism by which EBVaGC benefits from immunotherapy, but there are still many EBVaGC patients who are ineffective to neoadjuvant immunotherapy. Therefore, it is necessary to clarify the exact impact and potential mechanisms of EBV infection on the efficacy of ICB.

### 2.4 TMB

The TMB was defined as the count of non-synonymous mutations per megabase of the tumor genome reflecting tumor immunogenicity (85). The somatic TMB may lead to the formation of new antigens, and the presence of additional antigens enhances the probability that tumor cells will be detected by immune cells that infiltrate the tumor (86), thereby activating T-cell-mediated anti-tumor responses (87). Thus, medications stimulating T-cell activation, such as monoclonal antibodies targeting PD-1 or PD-L1, might offer potent anticancer treatment, especially for individuals with an elevated TMB. Several studies have demonstrated a favorable association between TMB levels and response to ICIs in different types of tumors (88–90). As a result, in June 2020, pembrolizumab was approved by the FDA for all solid tumors with high tumor mutational load (TMB-H). However, the TMB threshold used to screen for immunotherapy efficacy has led to different results in different clinical trials.

A TMB ≥ 10 mutations/megabase is a clinically significant cut-off point (91). In the Keynote-158 study, investigators used pembrolizumab to treat people with solid tumors showing an advanced high-TMB (TMB ≥ 10 mut/Mb). Twenty-four (10.3%) patients with GC were included. The results of the study found an ORR of 29% in patients with TMB-H tumors, compared to only 6% in patients who were non-TMB-H. Exploratory outcome analysis showed a correlation between TMB, and the clinical results of first-line pembrolizumab treatment and pembrolizumab combination chemotherapy after adjusting for CPS (ORR, PFS, and OS; all *P* < 0.05); those cases that had a TMB ≥ 10 mut/Mb and were managed with pembrolizumab demonstrated better clinical benefits (ORR, PFS, and OS) (92). Two additional studies reached the same conclusion, but with different TMB thresholds of 12 mut/Mb and 20 mut/Mb, respectively. A phase Ib/II clinical trial conducted by F. Wang et al. revealed that for AGC patients who failed first-line chemotherapy receiving Toripalimab monotherapy, regardless of the expression level of PD-L1, the OS of patients with ≥ 12 mut/Mb was significantly better than that of patients with < 12 mut/Mb (32). The findings of Zhang et al. showed that in advanced, resectable MSI-H gastrointestinal tumors, patients with a high TMB (TMB ≥ 20 mut/Mb) responded well to neoadjuvant ICIs, regardless of PD-L1 levels. In the KEYNOTE-061 trial, pembrolizumab improved outcomes in patients with TMB ≥ 175 mutations/exome (whole exome sequencing assessment) compared to chemotherapy (19). The TMB exhibited significant associations with ORR, PFS, and OS (all, *P* < 0.05) in the pembrolizumab group, whereas no notable correlations were detected in the chemotherapy group.

The above experimental data suggest that the TMB status in GC may serve as a predictive biomarker for pembrolizumab efficacy. However, several factors limit its reliability. Heterogeneity: it should be noted that the TMB cutoff value varies across different tumor types. Therefore, it is necessary to determine the TMB cutoff in GC to establish its value as a predictive biomarker. Gene fusion: conventional TMB assays, such as whole exome sequencing (WES) or targeted panels, may not effectively capture gene fusion events, especially those in non-coding regions or involving complex rearrangements. Gene fusions may serve as oncogenic drivers in tumor progression (e.g., *CLDN18-ARHGAP* fusion in GC) (93) and can generate neoantigens. However, the impact of gene fusions on immunotherapy response is not captured by TMB (94), which may lead to an underestimation of immunogenic potential. Moreover, fusion-driven tumors (e.g., NTRK fusions) exhibit a distinct immune microenvironment, where high TMB may not reliably predict treatment efficacy but instead shows stronger correlation with targeted therapy response. Clonality: TMB only reflects the total mutational burden, encompassing both clonal (truncal) and subclonal mutations, yet clonal mutations alone may more reliably indicate immunogenicity. Subclonal mutations may contribute to immune escape due to spatial heterogeneity. The heterogeneity of mutational profiles between primary and metastatic sites may compromise the accuracy of TMB assessment based on a single biopsy. Quality of mutations: not all mutations are capable of generating effective immunogenicity. Both nonsynonymous mutations (which may generate neoantigens) and synonymous mutations (which do not alter the amino acid sequence and lack immunogenicity) are counted in TMB, yet only the former may influence immunotherapy response (95). Driver mutations (e.g., *TP53*, *KRAS*) may indirectly modulate immune response by altering the tumor microenvironment (96, 97). Although passenger mutations inflate TMB values, most fail to elicit effective immune responses due to issues such as protein stability or impaired MHC binding. TMB cannot distinguish their respective contributions. Mutation type: indels and frameshift mutations may generate highly immunogenic neoantigens, but certain detection methods (e.g., targeted panels) exhibit limited sensitivity for these mutation types. Logistic issue: the TMB is also affected by a variety of factors, such as tumor type, detection method, and analysis technique. For instance, WES covers the entire exonic region but is costly, while targeted panels only interrogate a limited set of genes (98), potentially leading to TMB underestimation. In bioinformatics analysis, both variant calling algorithms and germline mutation filtering criteria can significantly impact the final TMB calculation.

Currently, research is gradually emerging on combining TMB with other biomarkers to predict the effectiveness of immunotherapy. For instance, recent studies suggest that combining TMB with T-cell-inflamed gene expression profile (GEP) can achieve superior predictive performance. In the KEYNOTE clinical dataset, it has been demonstrated that patients with solid tumors, including GC, who exhibit both high TMB (TMB-H) and high GEP (GEP-H), achieve the highest ORR (99). The combination of TMB and TIDE has demonstrated robust predictive efficacy in breast cancer, lung adenocarcinoma, and hepatocellular carcinoma (100–103), with future studies planned to explore its predictive potential for immunotherapy response in GC. Moreover, genomic and transcriptomic factors, such as novel antigen presentation by MHC-I and II complexes, can predict ICI outcomes (87, 104). Currently, there is a lack of validation for such models in GC-specific cohorts. For GC patients who have completed immunotherapy clinical trials (e.g., ATTRACTION-4, KEYNOTE-062), retrospective extraction of transcriptomic data from archived samples can be performed to validate the predictive efficacy of the aforementioned models. Additionally, prospective randomized controlled clinical trials can be conducted to further evaluate the predictive capability of these models. It is important to note that the molecular heterogeneity of GC (e.g., EBV-positive type, genomically stable type) may affect the generalizability of the models. Subgroup analyses (e.g., stratified by PD-L1 CPS, MSI, and EBV status) can be conducted to validate the universality of the models. The results are promising, as they may provide new directions for the development of biomarkers for GC immunotherapy.

## 3 Novel biomarkers

**Figure 3:**
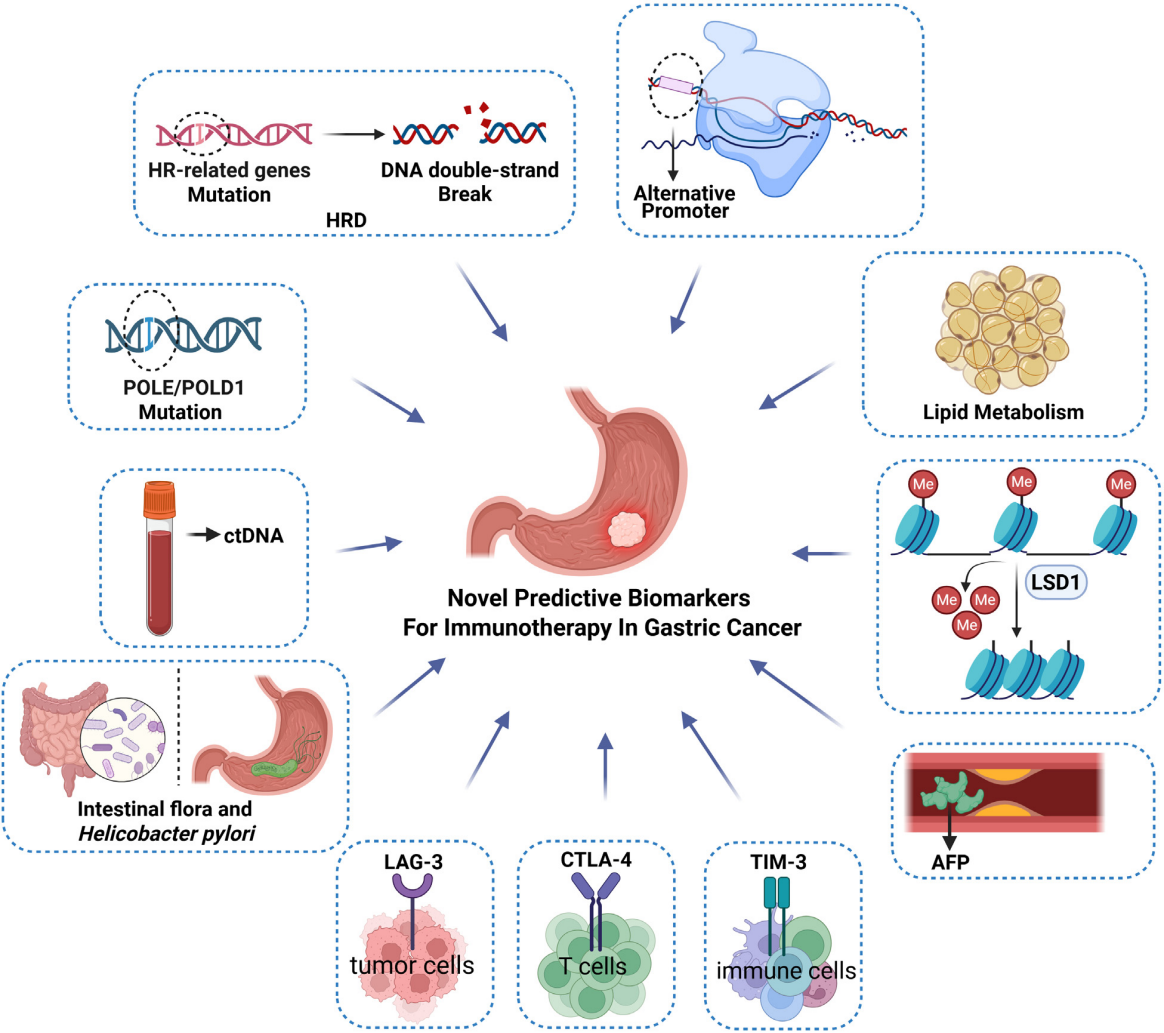
Novel biomarkers possibly predicting the efficacy of neoadjuvant immunotherapy for GC include POLE/POLD1 mutations, circulating tumor DNA (ctDNA), intestinal flora/*Helicobacter pylori*, cytotoxic T lymphocyte antigen-4 (CTLA-4), T cell immunoglobulin and mucin domain-containing protein 3 (TIM3), alpha fetoprotein (AFP), lysine-specific histone demethylase 1 (LSD-1), lipid metabolism and alternate promoters (AP) and homologous recombination deficiency (HRD).

A lack of reliable predictive biomarkers is one of the biggest issues in ICI therapeutics. In the past, a single immune-specific marker has been the focus of biomarker research, but the approved PD-L1, TMB and MSI-H cannot completely screen out all people who benefit from new adjuvant immunotherapy. Therefore, novel and reliable biomarkers for population screening are urgently required (**Figure 3**).

### 3.1 POLE/POLD1mutations

POLE/POLD1 mutations are thought to be linked to a high TMB, an enhanced tumor immune reaction and a better response to ICIs (105). It has been shown that POLE and POLD1 mutations are able to be used as stand-alone biomarkers for anticipating the efficacy of pan-cancer immunotherapy, and that they are also markers of a poor prognosis (106–108). Zhu et al. found that patients with GC and *POLE/POLD1* mutations typically exhibit an acquired immune resistance to the TME, with an increase in PD-L1 expression and an elevation of TMB (109). The above suggests that mutations of the *POLE/POLD1* gene can be used as a biomarker to improve the clinical efficacy of neoadjuvant immunotherapy in people with GC; identical results were obtained in NCT03012581. In this trial, Rousseau et al. prospectively evaluated the efficacy of nivolumab monotherapy in patients with advanced *POLE/POLD1* mutated solid tumors. Two patients (9%) with GC were included in this trial. It was found that only tumors with selective pathogenic mutations in the catalytic site of the DNA-binding or nucleic acid exonuclease structural domains exhibited high mutational loads, high T-cell infiltration, and high response rates to anti-PD1 monotherapy (110).

### 3.2 Circulating tumor DNA

Given the variation in time and space of GC, blood-based predictive biomarkers from liquid biopsies have surfaced as a hopeful strategy for anticipating responses to immunotherapy (111). Circulating tumor DNA (ctDNA) is one widely studied biomarker in liquid biopsies (112). Jin et al. performed next-generation sequencing testing on 46 patients with metastatic GC treated with neoadjuvant PD-1 inhibitor immunotherapy. They showed that patients with a > 25% decrease in the frequency of the largest variant allele in the ctDNA assay had a longer median PFS (7.3 m vs. 3.6 m; *P* = 0.0011) and higher ORR (53.3% vs. 13.3%). The study also found that patients with *TGFBR2*, *RHOA*, and *PREX2* mutations in the GC had significantly shorter PFS than those without mutations (113). Results from another study that included 61 patients with metastatic GC receiving neoadjuvant therapy with pembrolizumab showed that a decrease in the concentration of ctDNA in the sixth week of continuous ctDNA monitoring can be used to predict a benefit from neoadjuvant immunotherapy (73). Qiao et al. treated patients with unresectable advanced GC using neoadjuvant dendritic cells mixed with cytokine-induced killer cells (DC-CIK; an immune cell therapy) in combination with chemotherapy. They found that the frequency and number of ctDNA mutations were reduced in 19 patients (63.3%) after DC-CIK infusion. Reduced ctDNA mutation frequency was associated with improved PFS and OS (*P* = 0.001) (114). The above suggests that ctDNA testing is useful in anticipating the performance of individualized neoadjuvant immunotherapy in patients with GC. A prospective phase II clinical trial (NCT05594381) is currently underway to investigate the feasibility of ctDNA for evaluating the performance of PD-1 inhibitors in combination with a SOX regimen as a neoadjuvant therapy for locally progressive GC. Another aim of this study is to construct a model for evaluating the efficacy of treatment so as to clarify the applicability of the neoadjuvant immunotherapy for locally progressive GC. The results are eagerly anticipated.

Liquid biopsy offers advantages. Compared to other emerging biomarkers (such as TMB, PD-L1, or immune gene signatures), ctDNA testing exhibits higher sensitivity, enabling the detection of minimal residual disease (MRD) tumor burden and early recurrence. Moreover, from a practical standpoint, ctDNA’s blood-based detection offers dual advantages: enhanced clinical accessibility and serial monitoring capacity for tracking disease progression. However, it also has limitations as a screening approach for predictive biomarkers in GC immunotherapy. First of all, technical methods and sample quality limit the sensitivity of liquid biopsies, which may fail to detect tumor biomarkers that are at low concentration levels. Second, compared to other biomarkers such as TMB, PD-L1, or immune gene signature in tumor specimen, biomarkers in the blood may not be tumor-specific, leading to false-positive results. Therefore, given the significant clinical potential of ctDNA, more studies are needed to further improve the reliability of liquid biopsy, thus contributing to the screening of a GC population sensitive to immunotherapy.

### 3.3 Intestinal flora and *Helicobacter pylori*

Recent studies have begun to focus on the relationship between the gut microbiota, and tumor progression and treatment outcomes. This has been proposed as a potential biomarker for participating in the outcome of immunotherapy in solid tumors (115, 116). Although specific gut microbes play a role in GC progression and immunomodulation, insufficient evidence exists to support their potential as efficacy biomarkers in neoadjuvant immunotherapy for GC.

Recent research indicates that *H. pylori* infection may hinder the growth and anti-tumor functions of CD8^+^ T cells, foster the transformation of naive T cells into Tregs, and modulate the production of inflammatory mediators. These actions influence the TIME, dampen host immune reactions, and diminish the effectiveness of immunotherapy for GC (117–119). Therefore, patients with locally advanced GC should be aware of their *H. pylori* infection status before receiving neoadjuvant immunotherapy. However, forward-looking research exploring the relationship between *H. pylori* and prognosis in GC immunotherapy is lacking. In addition, the use of *H. pylori* as a predictive marker of immunotherapy efficacy needs to be thoroughly demonstrated (120).

### 3.4 Lymphocyte activation gene 3

LAG-3 is an inhibitory receptor on cell surfaces, which negatively regulates both CD8^+^ and CD4^+^T cell activity. It maintains immune system homeostasis under normal physiological conditions (121, 122), while LAG-3 and Treg cell interactions stimulate Treg activity, strengthen immune tolerance, and indirectly suppress dendritic cell (DC) function, thereby facilitating tumor cell immune escape. Consequently, LAG-3 is a promising therapeutic target in cancer immunotherapy, complementing the PD-1/PD-L1 pathway (123). Currently, the joint application of LAG-3 and PD-1 inhibitors is a topical research field. Kelly et al., in their innovative study, combined PD-1 and LAG-3 inhibitors with chemoradiotherapy for the neoadjuvant treatment of patients with gastroesophageal cancer (124). The authors reported that higher baseline PD-L1 (CPS ≥ 5) and LAG-3 expression was associated with superior pathological responses, with a Phase Ib clinical study also providing safety insights on combining PD-1 and LAG-3 for gastroesophageal cancer. But, in a Phase II study (RELATIVITY-060), combined nivolumab and the LAG-3 inhibitor relatlimab with chemotherapy for advanced G/GEJC failed to reach its primary endpoint (125). Therefore, more research is required to test the safety and effectiveness of PD-1 inhibitors when combined with LAG-3 inhibitors as an immunotherapy for patients with GC.

### 3.5 Cytotoxic T lymphocyte antigen-4

CTLA-4 is a co-inhibitory molecule on activated T and regulatory T cell surfaces (Tregs). It interacts with B7-1/B7–2 ligands on antigen-presenting cells, thereby inhibiting CD28-mediated signaling, which is responsible for T cell activation. Monoclonal antibodies targeting CTLA-4 have been shown to block its competition with CD28 for binding to B7, which in turn activates the CD28 signaling cascade and reduces immunosuppressive Treg cell populations in the TME (126). As the world’s first PD-1/CTLA-4 bispecific antibody, in the COMPASSION-15 (AK104-302) Phase III trial, cadonilimab showed significant efficacy in patients with different PD-L1 expression levels. In the trial, the proportion of individuals with PD-L1 CPS < 5 and CPS < 1 reached 49.8% and 23%, respectively, which was better reflected real-world patients. Therefore, these data confirmed the unique advantages of cadonilimab in individuals with low PD-L1 expression, and reflecting this, on September 30^th^, 2024, the National Drug Administration officially approved its combination with XELOX for the first-line treatment of unresectable locally advanced recurrent or metastatic G/GEJ adenocarcinoma.

### 3.6 T cell immunoglobulin and mucin domain-containing protein 3

TIM-3 is encoded by *HARVCR2* (127) and is an emerging target for cancer immunotherapy (128). Studies have shown that TIM-3 expression levels in GC tissue are significantly higher than in normal gastric mucosa tissue (129, 130), and positively correlated with PD-1/PD-L1 expression levels in GC tissue (131). Chen et al., reported that TIM-3 potentially promoted CD8^+^ T cell dysfunction in GC, manifested by decreased IFN-γ, perforin, and Granzyme B (GzmB) levels, but increased PD-1 and CTLA-4 levels (132). Although *HARVCR2* mRNA is elevated in most GC subtypes, it is more highly expressed in EBV-positive and MSI subtypes, as characterized by increased immune characteristics and higher immunotherapy responsiveness (73). Further research is required to examine the potential benefits of anti-TIM-3 inhibitors in patients with GC, and investigate their potential in combination with anti-PD-1/PD-L1 inhibitors.

### 3.7 Alpha fetoprotein

AFP has a wide range of biological functions, with previous research showing that it directly stimulated cancer cell proliferation and growth while also inhibiting apoptosis (133). AFP also inhibited monocyte differentiation to fully functional DCs and prevented them presenting foreign antigens to CD8^+^ lymphocytes via MHC signaling (134, 135). Furthermore, AFP down-regulated Toll-like receptor 4 expression on DCs and inhibited pro-inflammatory cytokine secretion, including interleukin-12 and tumor necrosis factor-α. These cytokines were shown to stimulate CD4^+^ and CD8^+^ lymphocyte production in immunotherapy (136). Additionally, AFP also induced ThCD4^+^ lymphocytes to differentiate into Tregs, thus negatively regulating immunotherapy. Zhang et al., reported that AFP levels predicted ICI efficacy in treating advanced GC, i.e., high baseline AFP levels were associated with reduced disease control rate (DCR) during ICI treatment and also shortened PFS and OS (137). However, the exact mechanisms whereby AFP levels affect ICI efficacy in patients with GC remain unclear and further research is needed.

### 3.8 Lysine-specific histone demethylase 1

In 2004, LSD-1 was characterized as the first histone demethylase (138); its expression was significantly elevated in GC and it promoted GC proliferation and metastasis (139–142). LSD-1 also mediates epithelial-mesenchymal transition (143) in GC via H3K4me2 demethylation, thereby promoting drug resistance, disease recurrence, and disease invasion and metastasis (144). Shen et al., reported that *LSD-1* deletion offset its immunosuppressive functions by reducing PD-L1 levels in exosomes and inhibiting its transport to other cancer cells, thereby restoring T cell killing functions in the GC microenvironment (145). Therefore, LSD-1 may function as a new immunotherapy target against GC, with the new LSD-1 inhibitor 5ac inhibiting mouse GC cell growth (146).

### 3.9 Lipid metabolism

An increasing body of evidence now indicates that reprogrammed energy metabolism has critical roles in GC progression (147). Therefore, more in-depth research on the metabolic changes in the GC TME may provide new markers/therapeutic targets for neoadjuvant GC immunotherapy. Yang et al., used database resources to identify eight genes associated with fatty acid metabolism, which correlated with GC prognosis outcomes. The authors developed ‘FRAS’, a model whereby FRAS scores effectively identified patients with GC who were likely to benefit from anti-CTLA-4 antibody immunotherapy. Among the eight genes, *RGS2* was significantly correlated with the TMB and CD8^+^ T cell infiltration in GC, suggesting that *RGS2* may be a potential target for future immunotherapy strategies toward GC (148).

### 3.10 Alternate promoters

On the one hand, the results obtained in previous clinical trials of PD-1/PD-L1 and TMB as screening indicators of whether GC patients can undergo immunotherapy have not been satisfactory. On the other hand, MSI-H and EBV GC patient population only accounts for a small proportion of all GC patients. Therefore, finding new positive predictive biomarkers and exploring negative biomarkers (that identify subjects with clear lack of benefit from specific therapy) are necessary. Promoters are cis-regulatory elements found upstream of transcription start sites, with > 50% of human genes having multiple promoters, i.e., AP (149). AP allow transcription to start at different transcription start sites, which then produces different 5’ untranslated regions and first exons, thereby enhancing mRNA and protein subtype diversity (150). Sundar et al., reported that patients with metastatic GC with high AP use expressed lower CD8A, GZMA, and PFR1 levels (151) (cytolytic T-cell activity marker (152, 153)), indicating that AP use in metastatic GC was inversely correlated with anti-tumor immunity. Subsequent studies reported that patients with advanced GC with higher AP activity showed higher CD8A and PRF1 levels (CTL surface markers) and lower LAG-3 and TIM-3 levels, thus creating an inhibitory immune microenvironment and a mechanism for GC immune escape (154).

### 3.11 Homologous recombination deficiency

Homologous recombination (HR) is a highly accurate DNA repair mechanism (155). On one hand, HRD induces DNA repair defects, leading to the accumulation of more mutations and the generation of neoantigens, which enhances tumor response to ICI (156). On the other hand, HRD may promote the formation of an anti-tumor immune microenvironment by increasing tumor-infiltrating lymphocytes (TILs) (157), suggesting its potential as a biomarker for predicting immunotherapy response. The study by Fan et al. revealed that HRD-positive GC patients exhibited significantly longer OS following ICI treatment compared to other GC patients. More importantly, the study also demonstrated positive correlations between HRD status and both TMB-H and MSI-H. Additionally, HRD-positive GC patients showed increased CD8^+^ T cell infiltration after ICIs treatment (158). These findings suggest that HRD has the potential to serve as a predictive biomarker for immunotherapy efficacy in GC.

## 4 Prospects

In this comprehensive review, we summarized some of the diverse markers used to evaluate immunotherapy efficacy, as documented in several clinical studies, among which, microsatellite instability and EBV associations were recognized as GC subtypes that could benefit the most from ICIs. However, few studies have investigated immunotherapy responses involving two other GC categories in the GC landscape, namely, chromosomal instability and genomic stability; therefore, more studies in these areas are warranted.

Nevertheless, each epitope marker also has its strengths and limitations. For example, temporal or spatial heterogeneity exists in PD-L1, delineating risk thresholds for markers whose measurements are continuous variables (e.g., TMB, PD-L1, etc.) is problematic, and the standardization of marker measurements or calculation methods is required. Therefore, it is difficult for a single biomarker to anticipate the performance of immunotherapy. The development of a mathematical model that includes all the key procedures of anti-tumor immunity to assess the immune status of an individual is a future trend.

One of the key reasons limiting the efficacy of ICIs in patients with GC is the immunosuppressive component of their TIME and the complex immune escape mechanisms involved. However, to date, little research has been conducted on the relationship between biomarkers and TIME, and their dynamic evolution during immunotherapy. An in-depth exploration of the effect of immunotherapy on GC TIME, an understanding of drug resistance mechanisms, the discovery of specific regulatory targets of GC TIME, and searching for more reliable, comprehensive, and dynamic biomarkers for population screening in an effort to bring immunotherapy for GC into the era of precision therapy, are required for the continued development of immunotherapy.
